# Exploring the Impact of Sensory Impairment

**DOI:** 10.1093/geroni/igaf122.1619

**Published:** 2025-12-31

## Abstract

In this session the association of sensory impairment, vision, hearing and olfaction are discussed including impact on caregiving, falls, cognition and cost.

